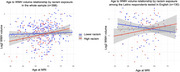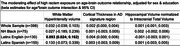# Racism as a Moderator of the Age‐Brain Relationship in a Multiethnic Cohort of Middle‐Aged Adults

**DOI:** 10.1002/alz70860_107289

**Published:** 2025-12-23

**Authors:** Dominika Seblova, Jessica Mazen, Justina F. Avila, Tiara M. Starks, Paris AJ Adkins‐Jackson, Patrick J. Lao, Adam Brickman, Jennifer J. Manly

**Affiliations:** ^1^ Second Faculty of Medicine Charles University, Prague, Czech Republic; ^2^ Columbia University Irving Medical Center, New York, NY, USA; ^3^ Taub Institute for Research on Alzheimer's Disease and the Aging Brain, New York, NY, USA; ^4^ Columbia University Mailman School of Public Health, New York, NY, USA

## Abstract

**Background:**

Structural racism contributes to increased stress and limits access to essential resources for health. Research on older adults suggests a link between structural racism and poorer cognition, which may explain racial and ethnic disparities in cognitive aging. However, there is a gap in evidence regarding how these factors influence brain outcomes.

**Method:**

We included 398 middle‐aged community‐dwelling adults (mean age=54.2, SD=12; 67% women; 10% non‐Latinx White; 19% non‐Latinx Black; 69% Latinx). An index of high racism exposure was developed from 3 self‐reported scales (Everyday Discrimination, Major Discrimination, Vigilance coping) and 3‐residential area factors (high poverty and two indicators of racial and ethnic segregation). Individuals in the top tertile of the continuous index were classified as having high exposure to racism. Outcomes included cortical thickness in Alzheimer's disease‐related regions, hippocampus volume (T1‐weighted MRI), and white matter hyperintensity (WMH) volume (T2 FLAIR MRI). We examined whether high racism exposure moderates the relationship between age and brain outcomes using linear regression models adjusted for sex and education, and for intracranial volume in analyses with hippocampal volume. Stratified models explored racial and ethnic differences.

**Result:**

Exposure to racism was highest among non‐Latinx Black adults (61%) and English‐speaking Latinx respondents (42%). Higher exposure to racism moderated the association of age with WMH volumes among Latinx respondents that chose test administration in English; the association between age and WMH volume was stronger among those exposed to higher racism compared to those exposed to lower racism (interaction: b=0.093 95%CI: 0.024;0.162). For all groups the moderation of between age and WMH volume showed a similar pattern, but none of the other associations reached statistical significance. For other outcomes, no reliable or sizable associations were observed.

**Conclusion:**

In this predominantly Latinx cohort, higher exposure to racism among English‐speaking Latinx individuals, due to greater interactions outside their communities, may explain the stronger association between racism and white matter hyperintensity (WMH) changes. These results suggest that racism may affect cerebrovascular health, potentially through chronic stress and inflammation, rather than directly influencing neuronal health.